# Supplementary material for: Mechanistically driven transnidal hemodynamic manipulations enhance simulated endovascular transvenous treatments for brain AVMs
Source: Commun Med (Lond). 2026 Apr 3;6:312. doi: 10.1038/s43856-026-01555-0 (PMC13216285; doi:10.1038/s43856-026-01555-0)
Supplement: Supplementary file 2 — Supplementary Information [file 43856_2026_1555_MOESM2_ESM.pdf]

SUPPLEMENTARY INFORMATION

ARTICLE | Communications Medicine

Mechanistically Driven Transnidal Hemodynamic Manipulations Enhance Simulated Endovascular Transvenous Treatments for Brain AVMs

Tarik F. Massoud[1,2,*], Bryce C. Vu[3], Kellen Vo Vu[4], Jeremy J. Heit[1,5], Siddhant Suri Dhawan[6]

[1]Division of Neuroimaging and Neurointervention, Department of Radiology, Stanford University School of Medicine, Stanford, CA, USA.

[2]Division of Interventional Neuroradiology, Department of Radiological Sciences, David Geffen School of Medicine at UCLA, Los Angeles, CA, USA.

[3]Arizona State University, Tempe, AZ, USA.

[4]MD Program, Weill Cornell Medicine, New York, NY, USA.

[5]Department of Neurosurgery, Stanford University School of Medicine, Stanford, CA, USA.

[6]Department of Bioengineering, Stanford University Schools of Engineering and Medicine, Stanford, CA, USA.

***Correspondence to:**
Professor Tarik F. Massoud, MD, PhD; Department of Radiology; Stanford University Center for Academic Medicine; Radiology, MC: 5659; 453 Quarry Road, Palo Alto; CA 94304; USA.
Tel: 650-7247026; Fax: 650-4985374; Email: tmassoud@stanford.edu

1. SUPPLEMENTARY FIGURES

SUPPLEMENTARY FIGURES

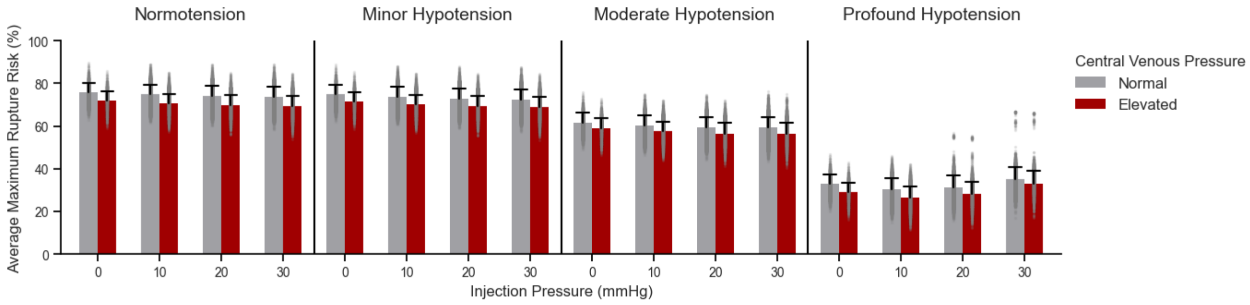

Supplementary Figure 1. Maximum rupture risk in the theoretical AVM model. Summary of average maximum rupture risk for each nidus, grouped by systemic blood pressure state (normotension, and mild, moderate, or profound hypotension), injection pressure (10 mmHg or 20 mmHg, or 30 mmHg), and CVP (normal of elevated). Error bars represent standard deviation.

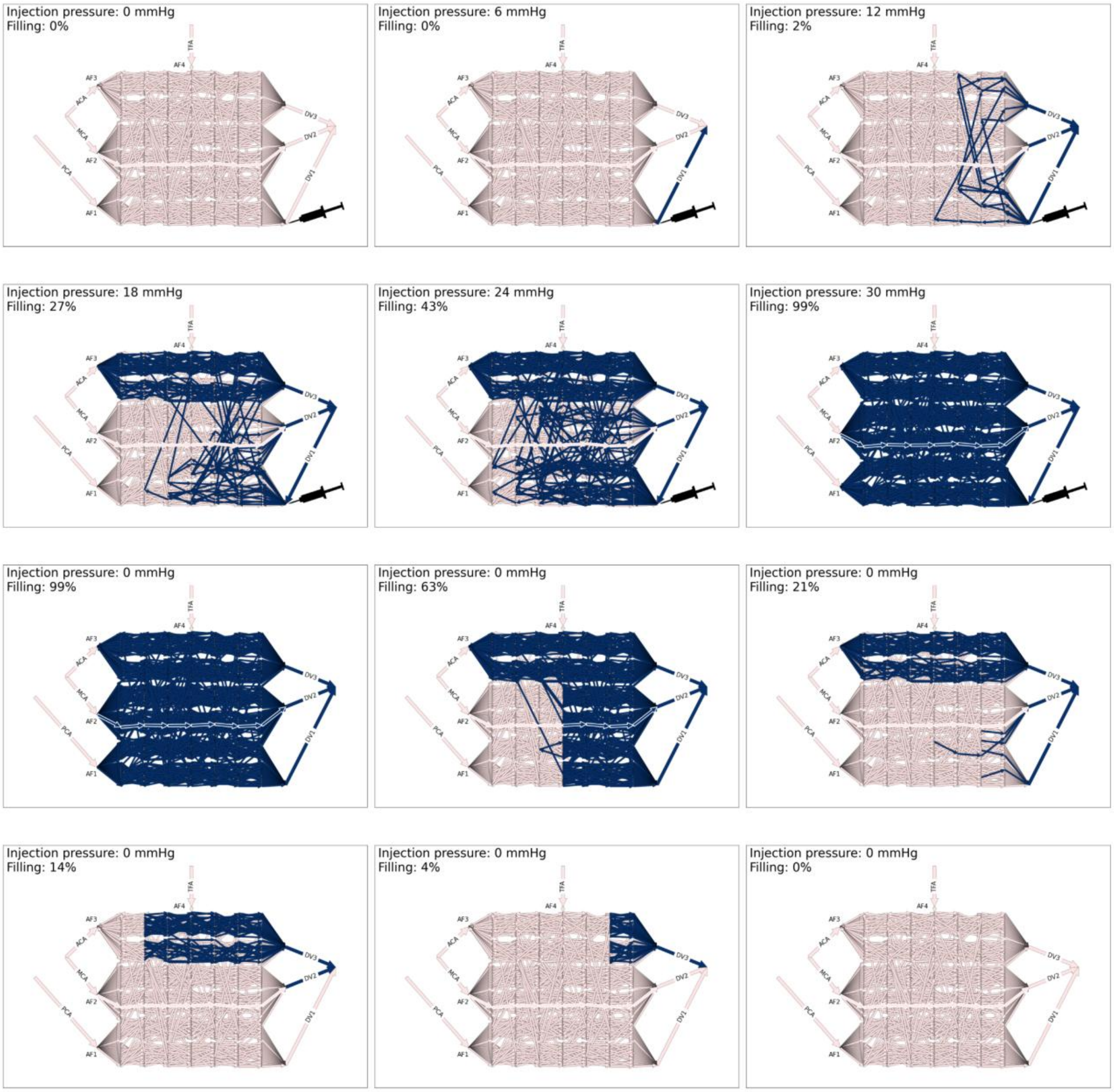

Supplementary Figure 2. Serial static illustrations for simulated nidus filling upon theoretical retrograde injection via DV3 in the theoretical AVM model. Top two rows demonstrate injection phase with sequential (left to right) snapshots of injectate spread upon incremental increase of 6 mmHg in injection pressures to a maximum of 30 mmHg, resulting in a maximum of 99% nidus filling. Bottom two rows demonstrate sequential phases of nidus emptying upon simulated termination of injection at the end of injectate delivery. See also Supplementary Movies 1-3 for dynamic examples of nidus filling and emptying.

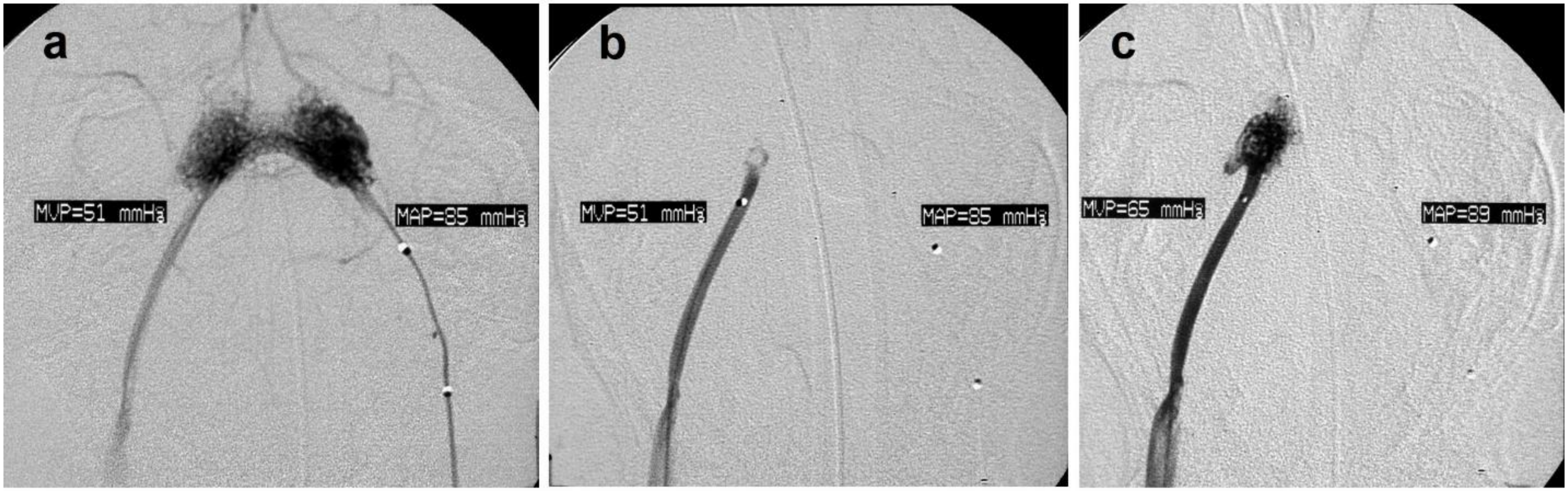

AF injection at syst-Normo

DV injection at syst-Normo: STAGE 1

DV injection at syst-Normo with AC: STAGE 2

Supplementary Figure 3. TRENSH simulations and hemodynamic studies using an *in vivo* AVM model showing successive frontal head and neck angiograms in pig #1 of Group 1. a, Superselective angiogram through the AF demonstrating baseline AF, nidus, and DV at systemic normotension; **b,** superselectively placed DV microcatheter for transvenous injection and angiogram shows minimal nidus retropermeation (Stage 1) at normotension; **c,** repeat transvenous angiogram at normotension with additional abdominal compression shows marginally greater nidus retropermeation (Stage 2). AC: abdominal compression; AF: arterial feeder; DV: draining vein; MAP: mean arterial pressure; MVP: mean venous pressure; syst-Normo: systemic normotension.

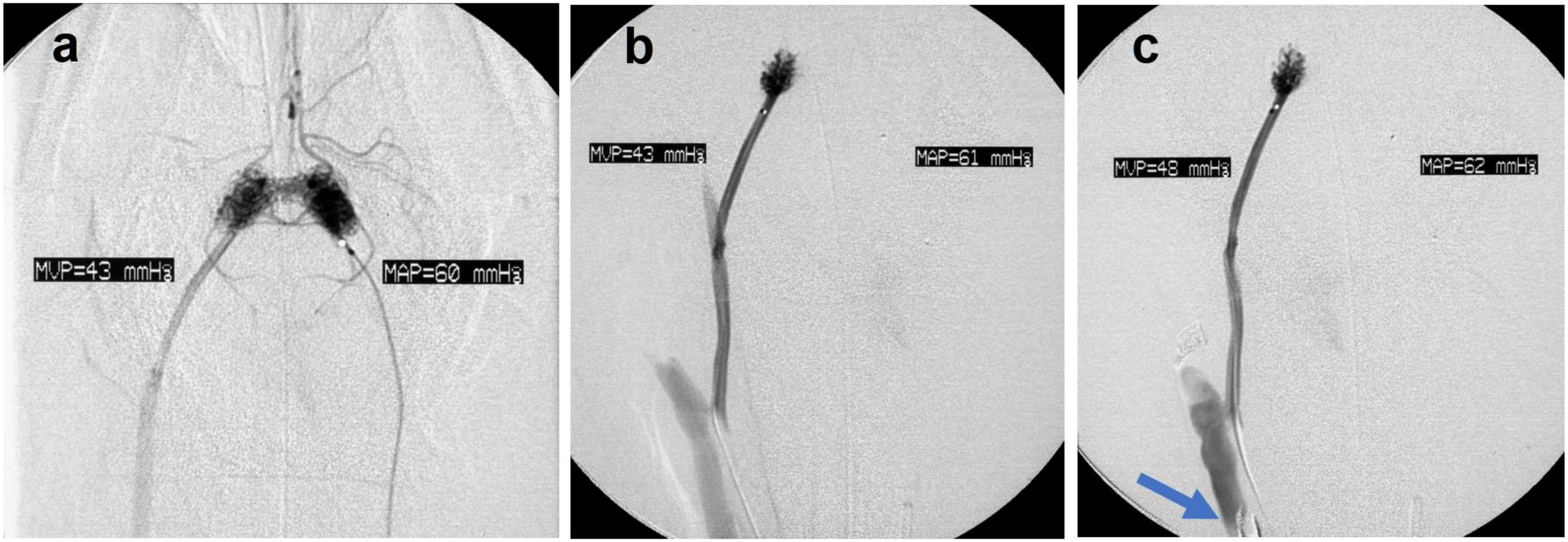

AF injection at syst-Normo

DV injection at syst-Normo: STAGE 2

DV injection at syst-Normo with vein clipping: STAGE 2

Supplementary Figure 4. TRENSH simulations and hemodynamic studies using an *in vivo* AVM model showing successive frontal head and neck angiograms in pig #3 of Group 1. a, Superselective angiogram through the AF demonstrating baseline AF, nidus, and DV at systemic normotension; **b,** superselectively placed DV microcatheter for transvenous injection and angiogram shows minimal nidus retropermeation (Stage 2) at normotension; **c,** repeat transvenous angiogram at normotension with additional internal jugular vein partial clipping (blue arrow) shows similar nidus retropermeation (Stage 2). AF: arterial feeder; DV: draining vein; MAP: mean arterial pressure; MVP: mean venous pressure; syst-Normo: systemic normotension.

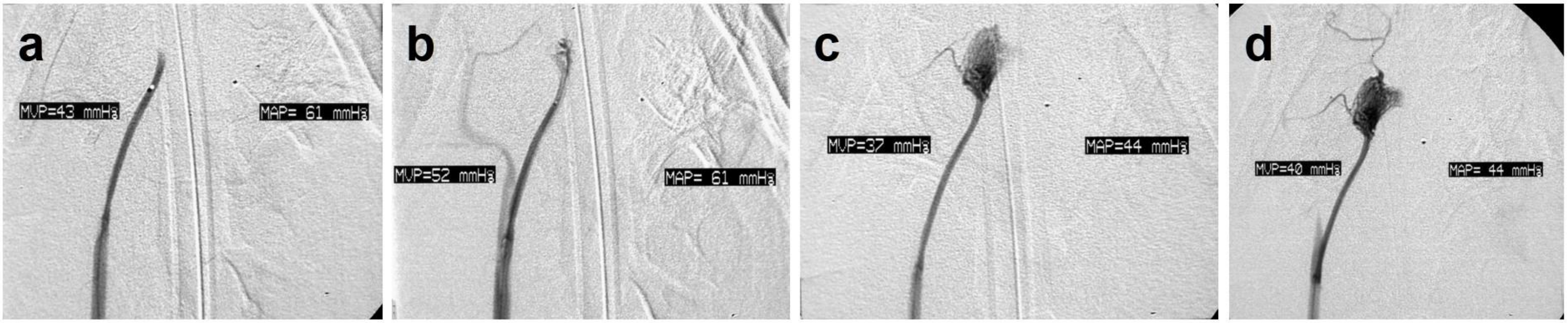

Supplementary Figure 5. TRENSH simulations and hemodynamic studies using an *in vivo* AVM model showing successive frontal head and neck angiograms in pig #5 of Group 2. a, Superselectively placed DV microcatheter for transvenous injection and angiogram shows minimal nidus retropermeation (Stage 1) at systemic normotension; **b,** repeat transvenous angiogram at normotension with additional internal jugular vein partial clipping (not seen on this and subsequent images) shows minimally greater nidus retropermeation (still Stage 2); **c,** repeat transvenous angiogram now with systemic hypotension and internal jugular vein clip removed shows greater nidus retropermeation (Stage 3); **d,** repeat transvenous angiogram with similar systemic hypotension but internal jugular vein clip reapplied shows greater nidus retropermeation (Stage 4). AF: arterial feeder; DV: draining vein; MAP: mean arterial pressure; MVP: mean venous pressure; syst-Hypo: systemic hypotension; syst-Normo: normotension.

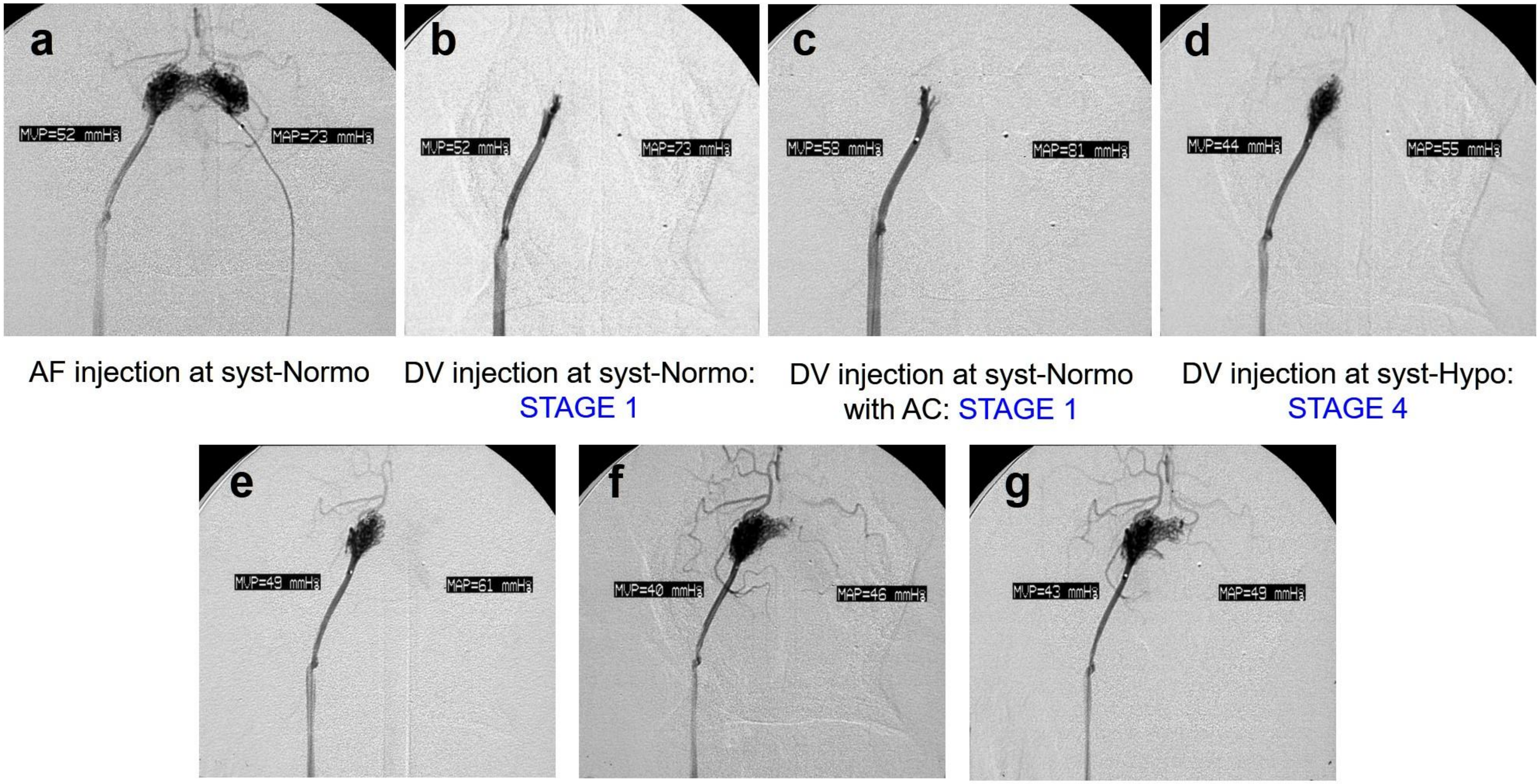

Supplementary Figure 6. TRENSH simulations and hemodynamic studies using an *in vivo* AVM model showing successive frontal head and neck angiograms in pig #6 of Group 2. a, Superselective angiogram through the AF demonstrating baseline AF, nidus, and DV at systemic normotension; **b,** superselectively placed DV microcatheter for transvenous injection and angiogram shows minimal nidus retropermeation (Stage 1) at systemic normotension; **c,** repeat transvenous angiogram at normotension with additional abdominal compression still shows minimal nidus retropermeation (Stage 1); **d,** repeat transvenous angiogram now with

systemic hypotension alone shows greater nidus retropermeation (Stage 4); **e,** repeat transvenous angiogram with similar systemic hypotension but now with abdominal compression shows marginally greater nidus retropermeation (but still Stage 4); **f,** repeat transvenous angiogram with deeper systemic hypotension and no abdominal compression shows greater nidus retropermeation (but still graded as Stage 4); **g,** repeat transvenous angiogram with deeper systemic hypotension and additional abdominal compression shows greater nidus retropermeation (Stage 5). AC: abdominal compression; AF: arterial feeder; DV: draining vein; MAP: mean arterial pressure; MVP: mean venous pressure; syst-Hypo: systemic hypotension; syst-Normo: normotension.

2. SUPPLEMENTARY TABLES

Supplementary Table 1. Vasculature parameters for theoretical AVM model construction, as per Hademenos, G. J., Massoud, T. F. & Viñuela, F. A biomathematical model of intracranial arteriovenous malformations based on electrical network analysis: theory and hemodynamics. *Neurosurgery*. **38**, 1005–1015 (1996).

**Vessel**	**R (cm)**	**L (cm)**	**R_v (dyne.s/cm^5)**
**Cardiovasculature**			
N13 – E_{SP} (superior vena cava)	0.750	10.0	2.817
E_{SP} – N1 (aortic arch)	1.000	10.0	0.891
N1 – N2 (subclavian artery)	0.350	10.0	59.393
**Head and neck vasculature**			
Neck and extracranial circulation			
N1 – N4 (common carotid artery)	0.350	10.0	59.393
N4 – N5 (external carotid artery)	0.200	10.0	557.042
N5 – N9	CP bed	CP bed	891267.681
N9 – N10	CP bed	CP bed	891267.681
N10 – N11	0.125	10.0	3650.632
N12 – N13 (jugular veins)	0.400	20.0	69.630
**Intracranial circulation**			
N4 – N6 (internal carotid artery)	0.250	20.0	456.329
N2 – N3 (vertebral artery)	0.150	25.0	4401.322
N6 – N7	0.100	10.0	8912.677
N7 – N8	CP bed	CP bed	891267.681
N8 – N11	0.125	10.0	3650.632
N11 – N12 (dural venous sinuses)	0.250	10.0	228.165
**AVM vasculature**			
**●Major arterial feeders**			
AF1 (posterior cerebral artery)	0.125	5.2	1898.329
AF2 (middle cerebral artery)	0.150	3.7	651.396
**●Minor arterial feeders**			
AF3 (anterior cerebral artery)	0.025	3.7	844208.748
AF4 (transdural feeding artery)	0.0125	3.0	10951897.268
**●Nidus vessels**			
Plexiform	0.01	0.05	445633.841
Fistulous	0.02	0.05	27852.115
**●Draining veins**			
DV1	0.250	5.0	114.082
DV2	0.250	5.0	114.082
DV3	0.250	5.0	114.082

AF: arterial feeder; AVM: arteriovenous malformation; CP: capillary; DV: draining vein; E_{SP}: electromotive force representing systemic pressure in mmHg; L: length; N: node; R: radius; R_v: vascular resistance.

Supplementary Table 2. Baseline electromotive force pressure values (in mmHg) for the different levels of systemic blood pressure, central venous pressure, and cardiac cycle conditions we simulated using the theoretical AVM model. Injection pressures at each location were added to the corresponding electromotive force.

Hypotension	CVP	Cardiac cycle	E_{SP}	E_{AF}	E_{DV}	E_{CVP}
Normotension	Normal	Intermediate	74	47	17	6
Minor			70	45	15	5
Moderate			50	32	12	5
Profound			25	15	8	4
Normotension	Elevated	Intermediate	74	47	22	12
Minor			70	45	19	10
Moderate			50	32	14	8
Profound			25	15	9	6
Normotension	Normal	Diastolic	74	43	15	6
Minor			70	44	14	5
Moderate			50	30	11	5
Profound			25	14	7	4
Normotension	Normal	Systolic	74	52	19	6
Minor			70	51	17	5
Moderate			50	34	13	5
Profound			25	17	9	4

CVP: central venous pressure
E_{SP}: electromotive force representing systemic pressure in mmHg
E_{AF}: electromotive force representing arterial feeder pressure in mmHg
E_{DV}: electromotive force representing draining vein pressure in mmHg
E_{CVP}: electromotive force representing central venous pressure in mmHg

Supplementary Table 3. Parameters and their respective values that were varied across the simulations on the 1,139 nidi of the theoretical AVM model.

Hypotension Level	CVP	Occlusion	Injection Location	If Injecting, Injection Pressure (mmHg)
Normotension	Normal	None	None	10
Minor	Elevated	AF1	DV1	20
Moderate		AF2	DV2	30
Profound		AF3	DV3	

AF: arterial feeder.
DV: draining vein.
CVP: central venous pressure.
