# Supplementary material for: Mechanistically driven transnidal hemodynamic manipulations enhance simulated endovascular transvenous treatments for brain AVMs
Source: Commun Med (Lond). 2026 Apr 3;6:312. doi: 10.1038/s43856-026-01555-0 (PMC13216285; doi:10.1038/s43856-026-01555-0)
Supplement: Supplementary file 3 — Description of Additional Supplementary Files [file 43856_2026_1555_MOESM3_ESM.docx]

**Description of Additional Supplementary Files**

Supplementary Movie 1- Video of sequential progressions of a tested strategy for TRENSH simulations in the theoretical bAVM model using moderate systemic hypotension, and a 30-mmHg injection through DV2

Supplementary Movie 2- Video of sequential progressions of a tested strategy for TRENSH simulations in the theoretical bAVM model using profound systemic hypotension, and a 20-mmHg injection through DV3

Supplementary Movie 3- Video of sequential progressions of a tested strategy for TRENSH simulations in the theoretical bAVM model using profound systemic hypotension, and a 30mmHg injection through DV3

Supplementary Movie 4- Cine angiogram at 30 frames/sec showing a close-up of the nidus region of the AVM model in pigs.
